# Early Diagnosis of Kidney Damage Associated with Tobacco Use: Preventive Application

**DOI:** 10.3390/jpm12071032

**Published:** 2022-06-24

**Authors:** Javier Tascón, Marta Prieto, Alfredo G. Casanova, Francisco J. Sanz, Miguel A. Hernández Mezquita, Miguel Barrueco Ferrero, Manuel A. Gomez-Marcos, Luis Garcia-Ortiz, Laura Vicente-Vicente, Ana I. Morales, on behalf of BIOTAB Team

**Affiliations:** 1 Toxicology Unit, Universidad de Salamanca, 37007 Salamanca, Spain; javiertascon_96@usal.es (J.T.); martapv@usal.es (M.P.); alfredogcp@usal.es (A.G.C.); amorales@usal.es (A.I.M.); 2 Institute of Biomedical Research of Salamanca (IBSAL), 37007 Salamanca, Spain; mhmezquita@gmail.com (M.A.H.M.); mibafe@telefonica.net (M.B.F.); magomez@usal.es (M.A.G.-M.); lgarciao@usal.es (L.G.-O.); 3 Group of Translational Research on Renal and Cardiovascular Diseases (TRECARD), 37007 Salamanca, Spain; 4 RICORS2040-Instituto de Salud Carlos III, 28029 Madrid, Spain; 5 Coria Primary Health Care Center, Health Service of Extremadura (SES), 10800 Coria, Spain; francsanz@hotmail.com; 6 Department of Pulmonology, University Hospital, 37007 Salamanca, Spain; 7 Unidad de Investigación en Atención Primaria de Salamanca (APISAL), Gerencia de Atención Primaria de Salamanca, Gerencia Regional de salud de Castilla y León (SACyL), Red de Investigación en Cronicidad, Atención Primaria y Promoción de la Salud (RICAPPS), 37007 Salamanca, Spain; 8 Department of Medicine, Universidad de Salamanca, 37007 Salamanca, Spain; 9 Department of Biomedical and Diagnostic Sciences, Universidad de Salamanca, 37008 Salamanca, Spain

**Keywords:** tobacco, early diagnosis, subclinical kidney damage, biomarkers

## Abstract

Although long-term smoking has been associated with chronic kidney disease, its effect on kidney function in early stages has not been clarified. Therefore, the proposed objectives were: (1) to identify subclinical kidney damage in smokers, through a panel of biomarkers; (2) to evaluate the progression of subclinical kidney damage after two years of consumption in these patients; and (3) study whether quitting smoking reduces kidney damage. A prospective study was carried out (patients recruited from a primary care centre and a clinical smoking unit). Kidney function was assessed using a panel of biomarkers and compared between smokers and non-smokers, taking into account potential risk factors for kidney damage. These results show, for the first time in the literature, the relationship between smoking and early (subclinical) kidney damage and provide a panel of biomarkers capable of detecting this condition (Neutrophil gelatinase-associated lipocalin, Kidney injury molecule-1, N-acetyl-beta-D-glucosaminidase, transferrin, and ganglioside-activating protein GM2). This study also indicates that subclinical damage is maintained when use continues, but can be reversed if patients stop smoking. The use of these biomarkers as diagnostic tools can be a preventive measure in the development of chronic kidney disease associated with smoking and in the prevention of acute events associated with potentially nephrotoxic pharmacological treatment in smokers. Trial registration number: NCT03850756.

## 1. Introduction

According to the World Health Organization, tobacco use leads to detrimental effects on public health and is responsible, directly and indirectly, for more than 7 million and 1.2 million deaths a year, respectively, worldwide [1].

In recent years, tobacco has been proposed as a risk factor for the progression of chronic kidney disease (CKD) in patients who suffer from pathologies related to kidney deterioration and in the general population [2,3,4]. The prevalence and incidence of end-stage CKD has steadily increased and is currently a global and devastating public health problem because of its medical, social, and economic consequences for patients and health systems [5]. Thus, health systems have been alerted to invest more in the identification of potentially avoidable preventive factors, such as tobacco use. 

A large cohort study conducted in 2016 [6] found that smoking is associated with a high glomerular filtration rate, and greater prevalence of proteinuria, suggesting a mechanism of hyperfiltration that could progress to CKD. Notably, in most published studies [7,8,9], renal function has been evaluated based on biomarkers that are not overly sensitive, such as the plasma creatinine or glomerular filtration rate. These biomarkers appear when kidney damage has been irreversibly established; thus, an early diagnosis is necessary, allowing intervention while the renal functional reserve has not yet been exhausted and the excretory function has not been compromised. 

So far, no exhaustive evaluation has been carried out on the effect of cigarette smoking on renal function in early-stage kidney disease with more sensitive biomarkers to assess whether tobacco alone can cause kidney impairment (at the subclinical level). Although the mechanisms involved in such damage have not been precisely described, it has been proposed that oxidative stress may play a relevant role [10]. Elucidating whether smoking cessation is related to an improvement in kidney function is relevant, as this may be a modifiable risk factor in the evolution of kidney damage. In this sense, an improvement in renal function has been associated with patients with CKD who quit smoking [7,11]; however, the effect of cessation on subclinical kidney disorders caused by tobacco use is unknown.

In view of these facts, this study proposed to evaluate the association between tobacco use and kidney damage by (1) determining a panel of early biomarkers to detect subclinical renal damage in smokers as well as oxidative stress biomarkers to evaluate its involvement in kidney damage; (2) evaluating the progression of subclinical kidney damage associated with tobacco after two years of consumption; and (3) assessing whether smoking cessation reduces subclinical renal damage.

## 2. Materials and Methods

The study protocol has been previously published [12].

### 2.1. Ethical Aspects

The study has been approved by the Clinical Research Ethics Committee (CEIC) of the Health Area of Salamanca (PI05/01/2018, 16 January 2018). All participants signed an informed consent form prior to the study in accordance with the Declaration of Helsinki and World Health Organization standards for observational studies [13]. During the development of this study, no alteration was required in the sanitary procedures for which the patients attended medical consultations. Patient data were treated confidentially in accordance with the provisions of current legislation on personal data protection and conditions contemplated by Act 14/2007 on biomedical research [14]. The patients were informed of the proposed objectives and benefits of the project, and they were free to withdraw from the study at any time.

### 2.2. Patients and Study Design

The patient recruitment procedure for each of the study’s objectives is explained below:

Objective 1: to evaluate the association between tobacco use and subclinical kidney damage using a panel of early biomarkers as well as the possible role of oxidative stress in the development of such damage.

A cross-sectional analysis using a prospective study’s baseline was conducted with patients recruited from the primary care centre, La Alamedilla (Salamanca), and a tobacco cessation clinic unit (University Hospital, Salamanca). 

The patients were divided into four groups based on their smoking habits and risk factors. Any person who had used tobacco either at the time of recruitment or during the previous year was considered a smoker. A person who had never used tobacco was considered a non-smoker. Diabetes mellitus, hypertension, and/or frequent use of non-steroidal anti-inflammatory drugs (>three days per week during the three months prior to recruitment) were considered risk factors for kidney disease. Considering these aspects, the experimental groups were distributed as follows: non-smokers with no risk factors (NS-NRF); non-smokers with risk factors (NS-RF); smokers with no risk factors (S-NRF); smokers with risk factors (S-RF) (Table 1).

Baseline urine samples were collected from the patients. A panel of biomarkers for kidney damage was determined for these samples. In addition, several oxidative stress biomarkers were analysed, as detailed later in Section 2. 

Objective 2: to evaluate the progression of subclinical kidney damage associated with tobacco after two years of consumption.

A prospective study was conducted with patients from the La Alamedilla health centre with a follow-up of two years. The patients remained in the same study group, implying that their smoking habits and risk factors had not changed in the two years.

The increase in the urinary biomarkers of kidney damage was calculated as the difference between the values obtained at two years and the baseline values.

Objective 3: to evaluate if smoking cessation reduces subclinical renal damage.

A cross-sectional analysis using the baseline of a prospective study was conducted with patients recruited from the La Alamedilla health centre’s primary care consultation and a tobacco cessation clinic unit at the University Hospital, Salamanca. 

To meet this objective, a new group of patients was included: former smokers with no risk factors (FS-NRF) (Table 1). Former smokers were defined as those patients who had been smokers but had stopped smoking at least one year before their inclusion in the study. To determine whether their cessation of tobacco use improved kidney function, biomarkers of kidney damage were evaluated in these patients as for the previous objectives. These results were compared with the groups that did not present risk factors (NS-NRF and S-NRF).

### 2.3. Inclusion and Exclusion Criteria

Patients who were of legal age, had signed the informed consent form, and did not meet any of the following exclusion criteria were included in the study. 

Patients experiencing any of the following conditions were excluded from the study: those with terminal illnesses; those who had been previously diagnosed with kidney damage; those who had been treated during the previous week or at the time of recruitment with aminoglycosides, cephalosporins, tetracyclines, amphotericin B, cisplatin, cyclosporine, foscarnet, acyclovir, cidofovir, radiological contrasts, or any other potentially nephrotoxic drug.

An encryption data system was established to protect the patients’ identities. The study data were collected and managed using the REDCap electronic data capture tools hosted at the University of Salamanca. A computerised database (Microsoft Office Excel 2016, Microsoft, Redmont, WA, USA) was created using the date of sample collection, general data (age and sex), anthropometric measurements (body weight, height, and body mass index), biochemical parameters (plasma creatinine and urea), risk factors for kidney damage (hypertension, diabetes mellitus, hypercholesterolaemia, smoking, and pharmacological treatments), and tobacco use (number of cigarettes/cigars consumed per day).

### 2.4. Sample Collection

Urine samples were collected from all patients at the time of consultation. On the same day, the samples were centrifuged (2000× *g*, 4 °C for 8 min), aliquoted, and stored at −80 °C until analysis.

### 2.5. Quantification of Kidney Damage Biomarkers in Urine Samples

A panel of kidney damage biomarkers was evaluated in the urine samples. Proteinuria was measured with the Bradford assay [15]. N-acetyl-β-D-glucosaminidase (NAG) activity was quantified using a commercial kit (“N-acetyl-β-D-glucosaminidase (NAG) assay kit”, Diazyme, Poway, CA, USA) following the manufacturer’s instructions. The ELISA technique was used for the following proteins (as indicated by the manufacturer’s instructions): neutrophil gelatinase-associated lipocalin (NGAL) (“Human NGAL ELISA Kit 036CE”, BioPorto Diagnostics, Hellerup, Denmark), kidney injury molecule 1 (KIM-1), (KIM-1 human ELISA Kit #ADI-900-226”, Enzo Life Sciences, Farmingdale, NY, USA), albumin (Human Albumin ELISA Quantitation Set E80-129” plus ELISA Starter Accessory kit E101” kit, Bethyl Laboratories, Montgomery, TX, USA), and transferrin (“Human Transferrin ELISA Quantitation Set E80-128” plus ELISA Starter Accessory kit E101” kit, Bethyl Laboratories, Montgomery, TX, USA).

The ganglioside GM2 activator protein (GM2AP) biomarker was determined by western blotting. Briefly, 21 μL urine was separated by 4–20% gradient polyacrylamide gel electrophoresis (4–20% Criterion TGX Stain-Free Protein Gel, Bio-Rad Laboratories, Hercules, CA, USA). Proteins were then electrically transferred to an Immun-Blot PVDF Membrane (Bio-Rad Laboratories, Hercules, CA, USA) and incubated with anti-GM2AP (own production), followed by horseradish peroxidase-conjugated secondary antibody and chemiluminescent detection (Clarity Western ECL Substrate, Bio-Rad Laboratories, Hercules, CA, USA) with a ChemiDoc MP Imaging System (Bio-Rad Laboratories, Hercules, CA, USA). The bands were quantified by densitometry analysis using Image Lab software (Bio-Rad Laboratories, Hercules, CA, USA). Integral normalisation was conducted by referring to the band quantification data of the same positive control loaded in each gel.

All biomarker values were factored by the urinary creatinine concentration to normalise the effect of urine concentration [16]. The urinary creatinine level required for the normalisation of all biomarkers was measured using a commercial Quantichrom Creatinine Assay Kit (BioAssay Systems, Haywar, CA, USA).

### 2.6. Tobacco Use Biomarker: Urinary Cotinine

To evaluate tobacco consumption, urine samples were analysed for cotinine (nicotine metabolite) concentration, for which the commercial kit “Cotinine ELISA kit” (Abnova corporation, Taoyuan City, Taiwan) was used. Data were normalised by the urinary creatinine concentration as explained previously.

### 2.7. Oxidative Stress Determination

In the urine samples of the patients included in the first objective, a panel of oxidative stress biomarkers was also evaluated, for which the following commercial kits were used: “OxiSelect TBARS Assay Kit STA-330” for MDA quantification (Cell Biolabs, Inc. San Diego, CA, USA); “OxiSelect Total Antioxidant Capacity Assay Kit STA-360” (Cell Biolabs, Inc., San Diego, CA, USA); “8-Isoprostane ELISA kit 516351” (Cayman Chemical, Michigan, WA, USA); and “Human Vanin RD-VNN1-Hu” (Reddot Biotech Inc., Kelowna, BC, Canada).

### 2.8. Temporal Evolution of Biomarkers Calculation

To evaluate the temporal evolution of kidney damage biomarkers over time, their absolute increase was calculated:

Biomarker increase = Biomarker (2 years) − Biomarker (basal).

### 2.9. Statistical Analysis

The percentages for all qualitative parameters were compared between groups using either Pearson’s chi-squared or Fisher’s exact test with Bonferroni post-hoc tests. The normality of the continuous quantitative data was evaluated with the Shapiro–Wilk (n ≤ 50) test or Kolmogorov–Smirnov test (n > 50). Pairwise comparisons were made using Student’s t-test (for normal data) and Mann–Whitney U test (for non-normal data). Three to four groups were compared using the Kruskal–Wallis test (for non-normal data) and Bonferroni correction (post-hoc test). In addition, the correlation between urinary levels of cotinine, biomarkers of kidney damage, and biomarkers of oxidative stress was analysed using Spearman’s rank correlation tests (for non-normal data) in the NS-NRF and S-NRF groups.

The criterion for significance was set at *p* < 0.05. All statistical analyses were performed using the IBM SPSS Statistics software version 20 (International Business Machines, Armonk, NY, USA). 

## 3. Results

### 3.1. Patients’ Characteristics

To achieve the three objectives, patients were grouped according to their smoking habits and the presence of risk factors, as detailed in Section 2 (Table 1). The characteristics of patients in each subgroup are shown in Table 2.

The groups were similar in terms of sex and weight. Patients’ age was higher in the groups that included risk factors (NS-RF and S-RF), which was expected because these pathologies (such as arterial hypertension and diabetes) occur more frequently in older patients. The lower estimated glomerular filtration rates observed in these two groups were likely to have been associated with risk factors but were independent of smoking habits. However, there were no differences in plasma creatinine levels between the groups. Regarding patients with risk factors, the non-smokers (NS-RF) group had a higher percentage of hypertensive patients, whereas smokers (S-RF) had a higher percentage of diabetics and non-steroidal anti-inflammatory drug consumption.

Finally, regarding tobacco use, patients without risk factors (S-NRF) stated that they smoked more cigarettes per day than those with risk factors (S-RF). As this information was provided by the patients—and may not have been entirely true in some cases—consumption was verified by measuring the biomarker cotinine, a metabolite of nicotine, in urine samples.

### 3.2. Objective 1: To Evaluate the Association between Tobacco Use and Kidney Damage

**Figure 1:**
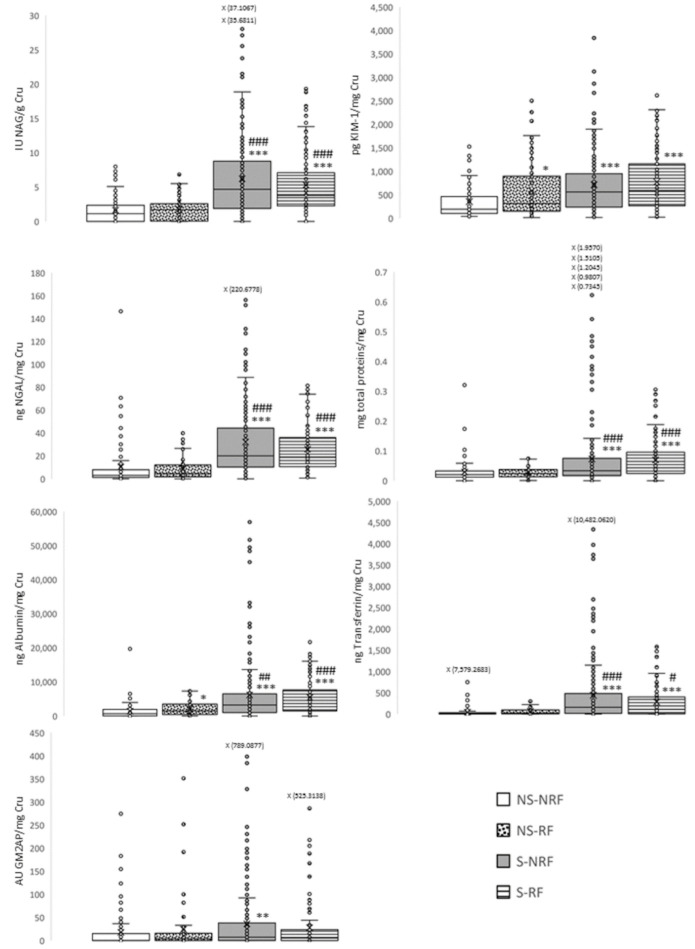
Association of tobacco consumption with kidney damage through a panel of urinary biomarkers. Data are presented in box plots. * *p* < 0.05; ** *p* < 0.01; *** *p* < 0.001 vs. NS-NRF. # *p* < 0.05, ## *p* < 0.01, ### *p* < 0.001 vs. NS-RF. NS-NRF: non-smokers, no risk factors; NS-RF: non-smokers with risk factors; S-NRF: smokers, no risk factors; S-RF: smokers with risk factors; AU, arbitrary units; Cru, urinary creatinine; GM2AP, ganglioside GM2 activator protein; KIM-1, kidney injury molecule 1; NAG, N-acetyl-β-D-glucosaminidase; NGAL, neutrophil gelatinase-associated lipocalin.

To study whether tobacco causes early alterations in kidney function, the urinary excretion of a panel of kidney damage biomarkers was compared between smokers and non-smokers with and without risk factors. Biomarkers capable of detecting alterations during the early stages of damage were used (Figure 1). Proteinuria, urinary NAG enzyme activity, and urinary excretion of NGAL and transferrin were statistically higher in smokers (S-NRF and S-RF) compared with non-smokers (NS-NRF and NS-RF). The urinary excretion of KIM-1 was significantly higher in all groups than in the NS-NRF group, with higher values in the smoker groups (S-NRF and S-RF). This pattern was repeated for albuminuria and showed significant differences between smokers (S-NRF and S-RF) and non-smokers (NS-RF). Finally, GM2AP excretion was only higher in smokers without risk factors (S-NRF).

The next step was to statistically determine whether patients who consumed more tobacco had greater renal impairment. Urinary cotinine levels were correlated with each evaluated biomarker of kidney damage (Table 3). To avoid confusion, this approach was followed only in groups without risk factors (NS-NRF and S-NRF). All biomarkers showed a statistically significant correlation, suggesting that renal impairment is directly proportional to tobacco consumption. Among these, NAG, NGAL, albumin, and transferrin showed the greatest significance, suggesting that these biomarkers are more sensitive for diagnosing kidney damage due to tobacco use.

**Figure 2:**
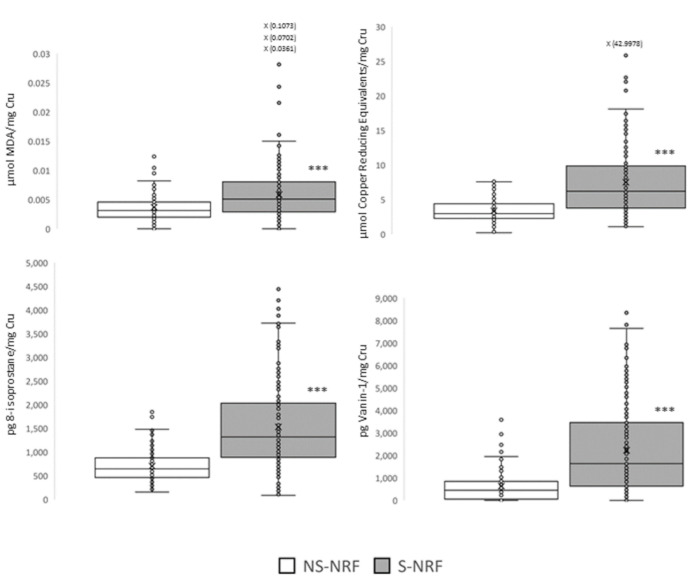
Evaluation of oxidative stress associated with tobacco consumption in groups without risk factors, through a panel of urinary biomarkers. Data are presented in box plots. *** *p* < 0.001 vs. NS-NRF. NS-NRF, non-smokers, no risk factors; S-NRF: smokers without risk factors. Cru, urinary creatinine; MDA, malondialdehyde.

To determine whether oxidative stress is involved in the nephrotoxic effects of tobacco, oxidative stress biomarkers were determined (Figure 2) in groups without risk factors, as the pathologies included among these factors could alter the redox state. The urinary excretions of all evaluated biomarkers were significantly higher in patients who smoked.

The correlation between these biomarkers and urinary cotinine levels was also determined (Table 4). There was a direct relationship between tobacco consumption and oxidative stress, which was similar for all measured biomarkers, suggesting that greater consumption led to greater oxidative alteration.

Regarding the association between biomarkers of renal damage and oxidative stress, it was observed that oxidative alteration is directly proportional to kidney damage, which is more evident when total antioxidant activity is evaluated. This suggests that tobacco induces oxidative stress and that the body activates antioxidant defence mechanisms to combat it.

### 3.3. Objective 2: To Evaluate the Progression of Subclinical Kidney Damage Associated with Tobacco after Two Years of Consumption

**Figure 3:**
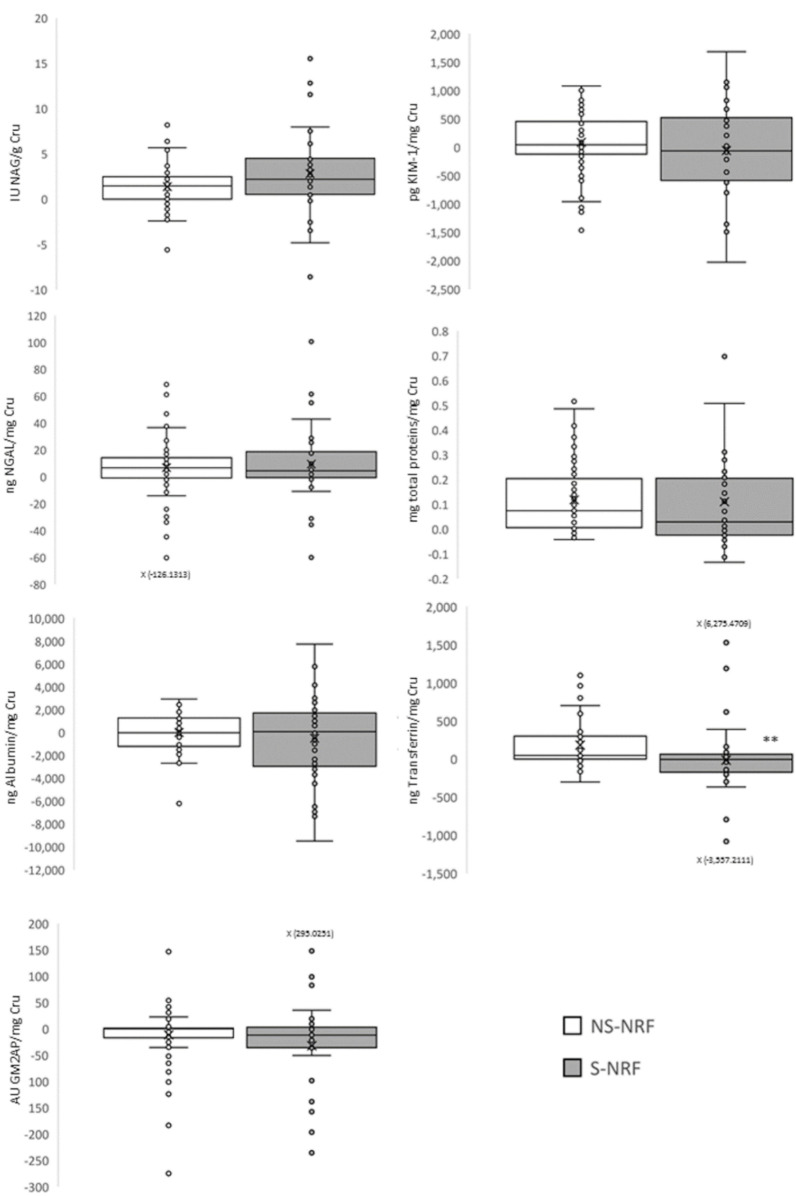
Evaluation of the progression of tobacco-associated subclinical kidney damage after two years of consumption through a panel of urinary biomarkers. Data are presented as absolute increase in box plots. ** *p* < 0.01 vs. NS-NRF. NS-NRF: non-smokers, no risk factors; S-NRF: smokers, no risk factors. AU, arbitrary units; Cru, urinary creatinine; GM2AP, GM2 ganglioside activating protein; KIM-1, kidney injury molecule 1; NAG, N-acetyl-β-D-glucosaminidase; NGAL, neutrophil gelatinase-associated lipocalin.

To study the renal effect of tobacco use after two years, the evolution of the panel of renal function biomarkers was evaluated by calculating their absolute increases in groups without risk factors (NS-NRF and S-NRF). No difference was observed between any biomarkers except for transferrin, which was lower in smokers after two years of use. These results indicate that the biomarker levels were similar to baseline levels (Figure 3). Therefore, the elevated levels of biomarkers in smokers compared with non-smokers observed in the previous objective persist over time.

### 3.4. Objective 3: To Evaluate If Smoking Cessation Reduces Subclinical Renal Damage

**Figure 4:**
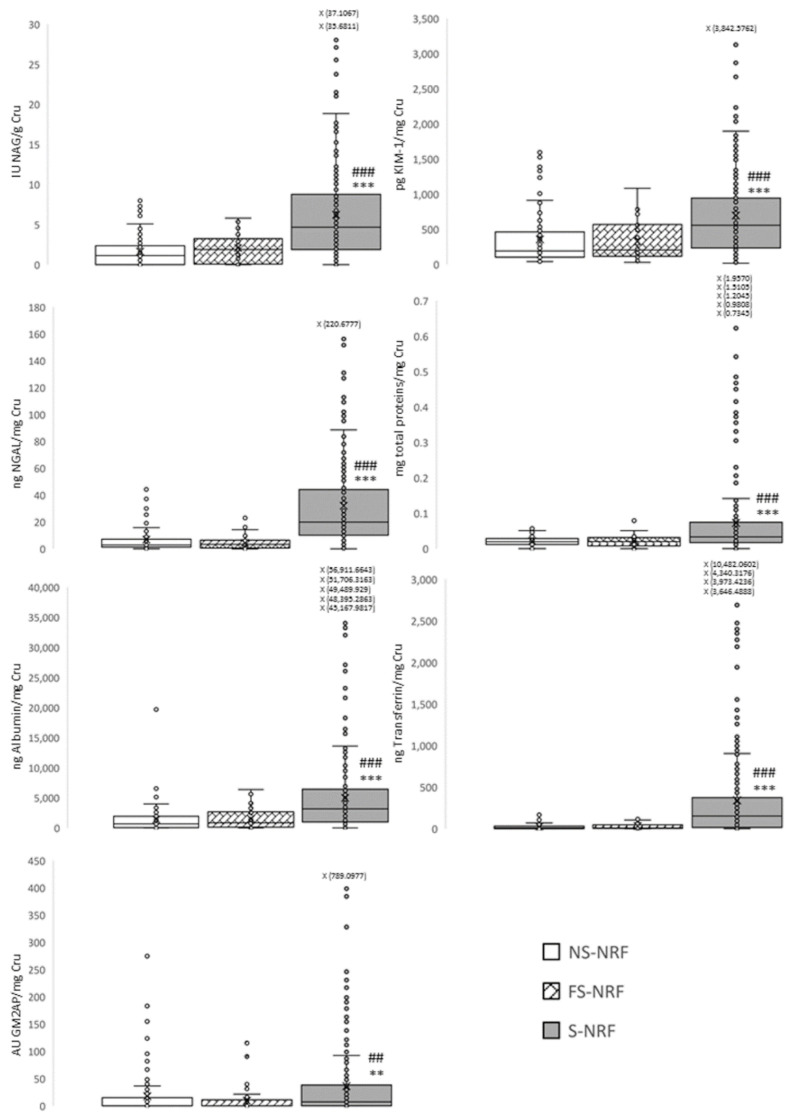
Effect of tobacco cessation on renal function through a panel of urinary biomarkers. Data are presented in box plot ** *p* < 0.01; *** *p* < 0.001 vs. NS-NRF. ## *p* < 0.01, ### *p* < 0.001 vs. FS-NRF. NS-NRF: non-smokers, no risk factors; S-NRF: smokers, no risk factors; FS-NRF former smokers, no risk factors; Cru, urinary creatinine; GM2AP, GM2 ganglioside activating protein; KIM-1, kidney injury molecule 1; NAG, N-acetyl-β-D-glucosaminidase; NGAL, neutrophil gelatinase-associated lipocalin.

To achieve this objective, a new study group was included (former smokers, no risk factors (FS-NRF)) in which a panel of biomarkers of kidney damage was determined and compared with other groups of non-smokers and smokers without risk factors (NS-NRF and S-NRF, respectively). Again, only groups without risk factors were included so that the results obtained could be attributed more directly to tobacco consumption and not to other factors. For all the analysed biomarkers (Figure 4), it was observed that the behaviour of the group of former smokers was comparable to that of non-smokers. Thus, the urinary excretion observed for all biomarkers was statistically higher in smokers than in the other two groups. This suggests that smoking cessation leads to an improvement in kidney function; therefore, the damage caused by tobacco is reversible.

## 4. Discussion

Although long-term smoking has been associated with CKD [17], the effect of cigarette smoking on renal function in early-stage kidney disease has not been clarified. Our results demonstrate the relationship between smoking and early (subclinical) kidney damage through a panel of urinary biomarkers, a first in the literature. The use of these biomarkers as diagnostic tools can be a preventive measure against the development of smoking-related CKD and acute events resulting from smokers’ treatment with potentially nephrotoxic drugs. This study also implies that subclinical damage is sustained over time when consumption continues but can be reversed if smoking is stopped.

Smoking-related renal injury shares pathophysiological mechanisms with diabetic nephropathy and hypertension, such as insulin resistance and increased sympathetic nervous activity [18], which is why an increase in albuminuria, proteinuria, and hyperfiltration is observed in most studies on the relationship between tobacco consumption and kidney damage (alterations similar to those pathologies) [19,20,21]. Our study confirms these findings for smokers with risk factors and also demonstrates the effect of smoking on urinary albumin/protein excretion in subjects from the general population with apparently normal kidney function, as described in a study by Halimi et al. [22] conducted with 28,409 subjects. Our findings also demonstrate that the albuminuria excretion rate is correlated with the number of cigarettes smoked per day (measured as cotinine excretion), similar to previous studies [23].

In recent decades, several biomarkers of kidney injury have been identified in urine and blood to address the well-recognised limitations of creatinine as an indirect biomarker of kidney damage. These biomarkers are either produced directly by the kidney or accumulate because of tubular cell dysfunction after kidney injury. Our results indicate an increase in the urinary excretion of NGAL, KIM-1, NAG, transferrin, and GM2AP, confirming subclinical damage in smokers (with and without risk factors). When the initial kidney injury is induced in the epithelial cells of the renal tubules, subtle changes occur, releasing specific proteins such as NGAL, KIM-1, or NAG into the urine and systemic circulation [24,25,26]. Some studies have also shown the utility of these biomarkers in the early diagnosis of diseases such as diabetic nephropathy, whose pathophysiological mechanism is similar to that caused by tobacco [27,28,29]. In contrast, urinary transferrin has been shown to be a biomarker capable of identifying subclinical tubular alterations and, therefore, pre-emptively identifying subpopulations of oncological and cardiac patients at risk of nephrotoxicity [30]. GM2AP and NGAL have also been shown to increase in urine owing to defective tubular handling of this protein as a consequence of the action of gentamicin [31,32]. 

Based on the evidence from these studies, our results suggest that the urinary excretion of these biomarkers can indicate early damage to the renal tubules. To the best of our knowledge, this is the first time that tobacco use has been associated with subclinical damage at the tubular level. It has been established that subclinical damage can create a condition of susceptibility that degenerates into acute kidney damage in certain circumstances; for example, when the patient is treated with potentially nephrotoxic drugs (such as aminoglycosides and platinum compounds) or is subjected to diagnosis tests with contrast media [30,33,34,35]. Therefore, the detection of this condition in smokers is a preventive strategy against exposure to renal toxicity. 

In contrast, the correlation between the excretion of these biomarkers and cotinine suggests a relationship between the level of consumption and the degree of kidney damage. Although this study has a limitation in that each smoker was different both in terms of the time of consumption and the number of cigarettes smoked per day, cotinine, as a biomarker of recent consumption, supports our argument.

The principal determinants of CKD development and progression are cardiometabolic risk factors, such as age, elevated blood pressure, dyslipidaemia, obesity, diabetes, and smoking [36]. It has also been shown that the risk of kidney failure is three to four times higher in smokers with risk factors (hypertension, diabetes mellitus, and cardiovascular disease) [7]. However, the effect of tobacco on the possible evolution of subclinical damage has not yet been studied. Our results indicate that the levels of biomarkers are maintained over time and, therefore, subclinical damage persists. Several studies have revealed that sustained tubular damage after acute kidney injury affects renal repair and results in gradual progression to CKD [37,38]. In this context, it is possible that this mechanism is involved in the development of CKD in smokers, and the sustained increase in biomarkers is indicative of a chronic process that would later degenerate into CKD. Nevertheless, our results are limited by the short study duration (two years) and should be confirmed using a long-term study.

Oxidative stress is one of the main mechanisms involved in kidney damage associated with tobacco. Chronic exposure to nicotine and free radicals in tobacco smoke has been shown to increase oxidative stress biomarkers [10,39]. Our results not only support these studies but also correlate this mechanism with an increase in the biomarkers of early kidney damage. Although it has been shown that antioxidant enzyme concentrations decrease as renal failure progresses [40], our results indicate a positive correlation between biomarker excretion and total antioxidants. In our study, antioxidants may have played a protective role that justified the subclinical state and the absence of renal alterations.

Finally, in relation to the cessation of tobacco consumption, there is evidence indicating that it is one of the most effective measures to prevent the progression of renal failure [8,9,41]. In a study on patients with diabetic nephropathy, disease progression was observed in 53% of smokers, but only in 33% of former smokers and 11% of non-smokers [42]. It is plausible to assume that this is also true for patients without apparent kidney disease. Pinto-Sietsma et al. [23,43] found that the risk of microalbuminuria was lower in former smokers but significant in smokers. Our results are encouraging, as they show that quitting smoking reverses kidney damage and prevents the possible development of CKD, which could constitute another health benefit as argued in cessation programmes.

## 5. Conclusions

In conclusion, the novelty of our results lies in presenting evidence of subclinical (possibly tubular) kidney damage in tobacco users. This study provides a panel of biomarkers capable of detecting this condition that can be used to implement preventive measures against the development of CKD and possible acute events derived from potentially nephrotoxic treatment in smokers.

## Figures and Tables

**Table 1:** Patient groups established in the study design. The X indicates that the patients in the group meet this criterion, that is, they are smokers or present at least one of the risk factors: diabetes mellitus, hypertension, and/or frequent use of non-steroidal anti-inflammatory drugs.

Group	NS-NRF	NS-RF	S-NRF	S-RF	FS-NRF
Smoker			X	X	
Former Smoker					X
Risk factor		X		X	

NS-NRF: non-smokers, no risk factors; NS-RF: non-smokers with risk factors; S-NRF: smokers, no risk factors; S-RF: smokers with risk factors; FS-NRF: former smokers, no risk factors.

**Table 2:** Descriptive characteristics of the patients included in the study.

	Non-Smokers		Smokers		Former Smokers
	No Risk Factors (n = 105)	Risk Factors (n = 80)	No Risk Factors (n = 194)	Risk Factors (n = 114)	No Risk Factors (n = 46)
Group Name	NS-NRF	NS-RF	S-NRF	S-RF	FS-NRF
Gender (female/male %)	43.8/56.2 _a_	51.2/48.8 _a_	53.6/46.4 _a_	39.5/60.5 _a_	63.0/37.0 _a_
Age (years, mean ± SEM)	53.28 ± 1.40	67.44 ± 1.18 ***	49.76 ± 0.77 ^###^	57.35 ± 0.96 ^###&&&^	54.83 ± 1.57 ^###^
Weight (kg, mean ± SEM)	72.35 ± 1.23	71.41 ± 1.28	72.62 ± 1.18	77.69 ± 1.42 *^#^	71.97 ± 2.05
Height (cm, mean ± SEM)	166.55 ± 0.92	160.88 ± 1.13 **	166.28 ± 0.64 ^##^	164.88 ± 0.85	166.02 ± 1.04
BMI (kg/m^2^, mean ± SEM)	26.05 ± 0.39	27.55 ± 0.37	26.11 ± 0.36 ^#^	28.41 ± 0.45 **^&&&^	26.00 ± 0.60 ^$$$^
Plasma creatinine (mg/dL)	0.83 ± 0.02	0.80 ± 0.02	0.79 ± 0.01	0.82 ± 0.02	0.81 ± 0.02
eGFR CKD-EPI (mL/min/1.73 m^2^, mean ± SEM)	94.03 ± 1.37	86.11 ± 1.49 **	97.14 ± 1.06	91.90 ± 1.44 ^#^	91.11 ± 1.76
Previous kidney disease (%)	0 _a_	0 _a_	0 _a_	9.8 _b_	0 _a_
Diabetes mellitus (%)	0 _a_	17.5 _b_	0 _a_	36.8 _c_	0 _a_
Arterial hypertension (%)	0 _a_	87.5 _b_	0 _a_	64.9 _c_	0 _a_
NSAIDs (%)	0 _a_	6.2 _b_	0 _a_	30.7 _c_	0 _a_
Number of cigarettes per day (n, mean ± SEM)	n.a.	n.a.	16.81 ± 0.66	14.78 ± 0.84 ^&^	n.a.
Urinary cotinine (mean ± SEM)	5.65 ± 2.81	3.42 ± 0.68 **	5289.83 ± 350.40 ^###^	2413.73 ± 276.75 ***^###&&&^	9.36 ± 7.82 ^#&&&$$$^

Quantitative variables: * *p* < 0.05, ** *p* < 0.01, *** *p* < 0.001 vs. NS-NRF; # *p* < 0.05, ## *p* < 0.01, ### *p* < 0.001 vs. NS-RF; & *p* < 0.05, &&& *p* < 0.001 vs. S-NRF; $$$ *p* < 0.001 vs. S-RF. Qualitative variables: groups with the same subscript letter are statistically similar. BMI, body mass index; eGFR, estimated glomerular filtration rate; n.a., not applicable; NSAIDs, non-steroidal anti-inflammatory drugs; SEM, standard error of the mean.

**Table 3:** Correlation between urinary levels of cotinine and early kidney damage biomarkers in smoking patients without risk factors. Data are expressed as Spearman’s correlation coefficient (ρ).

Urinary Biomarker	NAG	KIM-1	NGAL	Total Proteins	Albumin	Transferrin	GM2AP
Cotinine	0.488 ***	0.366 ***	0.473 ***	0.360 ***	0.447 ***	0.411 ***	0.218 ***

*** *p* < 0.001. GM2AP, ganglioside GM2 activator protein; KIM-1, kidney injury molecule 1; NAG, N-acetyl-β-D-glucosaminidase; NGAL, neutrophil gelatinase-associated lipocalin.

**Table 4:** Correlation between urinary levels of cotinine and early kidney damage biomarkers and urinary levels of oxidative stress biomarkers in smoking patients without risk factors. Data are expressed as Spearman’s correlation coefficient (ρ).

Urinary Biomarker	8-Isoprostane	Vanin-1	MDA	TAC
Cotinine	0.592 ***	0.524 ***	0.472 ***	0.569 ***
NAG	0.142 *	0.227 ***	0.163 **	0.240 ***
KIM-1	0.243 ***	0.301 ***	0.192 ***	0.238 ***
NGAL	0.108	0.303 ***	0.200 ***	0.349 ***
Total proteins	0.162 **	0.271 ***	0.196 ***	0.208 ***
Albumin	0.142 *	0.208 ***	0.196 ***	0.213 ***
Transferrin	0.250 ***	0.262 ***	0.181 **	0.194 ***
GM2AP	0.159 **	−0.009	0.054	0.159 **

* *p* < 0.05; ** *p* < 0.01; *** *p* < 0.001. GM2AP, ganglioside GM2 activator protein; KIM-1, kidney injury molecule 1; NAG, N-acetyl-β-D-glucosaminidase; NGAL, neutrophil gelatinase-associated lipocalin; TAC, total antioxidant capacity; MDA, malondialdehyde.

## Data Availability

The data presented in this study are available on request from the corresponding author.
